# Supplementary material for: MetaRibo-Seq measures translation in microbiomes
Source: Nat Commun. 2020 Jun 29;11:3268. doi: 10.1038/s41467-020-17081-z (PMC7324362; doi:10.1038/s41467-020-17081-z)
Supplement: Supplementary file 10 — Supplementary Data 7 [file 41467_2020_17081_MOESM10_ESM.zip › File2/Confidence_High_Taxonomy/155189_out.krona.html]

Javascript must be enabled to view this page.

members
magnitude
magnitudeUnassigned
count
unassigned
taxon
rank

155189\_out

4

2
4
superkingdom

4
phylum
1239

186801
class
4

186802
order
4

family
4
541000

1263
genus
4

41978
4
species

SRS011084\_contig\_number\_25972SRS015960\_contig\_number\_18867SRS021484\_contig\_number\_contig-100\_1684.154506SRS056259\_contig\_number\_29376
